# Gastroesophageal reflux disease and oral symptoms: A two-sample Mendelian randomization study

**DOI:** 10.3389/fgene.2022.1061550

**Published:** 2023-01-04

**Authors:** Shijing Jiang, Liang Zheng, Zhiwei Miao

**Affiliations:** ^1^ Department of Gastroenterology, Second Affiliated Hospital of Nanjing University of Chinese Medicine, Nanjing, China; ^2^ Jiangsu Provincial TCM Technology Engineering Research Center of Health and Health Preservation, Nanjing, China; ^3^ Department of Gastroenterology, Zhangjiagang TCM Hospital Affiliated to Nanjing University of Chinese Medicine, Zhangjiagang, China

**Keywords:** gastroesophageal reflux disease, mouth ulcers, toothache, loose teeth, bleeding gums, periodontitis, Mendelian randomization

## Abstract

**Background:** The association between Gastroesophageal reflux disease (GERD) and oral symptoms has been reported in observational studies, but the causality of GERD to oral symptoms remained unknown. We aimed to assess the causal effect of GERD on five oral symptoms (mouth ulcers, toothache, loose teeth, bleeding gums, and periodontitis) using the two-sample Mendelian randomization (MR) method.

**Methods:** Summary-level statistics for GERD and five oral symptoms were obtained from large-scale genome-wide association studies. Rigorous quality control of genetic instruments was conducted before MR analysis. Several analytical methods, including the inverse-variance weighted (IVW) method, MR-Egger regression, weighted median, maximum likelihood, and robust adjusted profile score (RAPS) were utilized, and the results of IVW were taken as the main results. The MR-Egger intercept test, Cochran’s Q test, and leave-one-out test were used as sensitivity analysis for quality control.

**Results:** After Bonferroni, IVW detected a significant effect of GERD on mouth ulcers (OR = 1.008, 95% CI = 1.003–1.013, *p* = 0.003), loose teeth (OR = 1.009, 95% CI = 1.005–1.012, *p* = 9.20 × 10^−7^), and periodontitis (OR = 1.229, 95% CI = 1.081–1.398, *p* = 0.002). Consistent patterns of associations were observed across several MR models and sensitivity analysis found little evidence of bias. Nominal significant associations were observed in toothache and bleeding gums (*p* < 0.05), and heterogeneity was detected.

**Conclusion:** Our MR analyses supported the positive causal effect of GERD on oral symptoms, especially for mouth ulcers, loose teeth, and periodontitis. Our findings might shed light on the mechanism of oral disease and might imply that oral care should be enhanced in patients with GERD.

## 1 Introduction

Gastroesophageal reflux disease (GERD) is a frequent clinical condition associated with upper gastrointestinal motility disorders that can cause stomach contents to reflux into the esophagus (Maret-Ouda et al., 2020). It is estimated that approximately 20% of the adult population in the Western world suffers from GERD (Delshad et al., 2020). Many complications can occur in GERD patients, including laryngopharyngeal, respiratory, and oral symptoms (Vaezi et al., 2018; Durazzo et al., 2020). The high prevalence and multiple complications of GERD can have a devastating impact on patients’ physical and mental health. Identifying the unfavorable outcomes in the GERD population in a causal fashion would aid in the prevention of GERD’s negative repercussions.

The association between GERD and oral symptoms is still in dispute. A majority of studies have found that patients with GERD may be more susceptible to oral soft and hard tissue symptoms, such as mouth ulcers, toothache, loose teeth, bleeding gums, and periodontitis (Song et al., 2014; Deppe et al., 2015; Watanabe et al., 2017). Some studies, however, only discovered associations between GERD and mucosal lesions, but not dental symptoms (Di Fede et al., 2008). The conflicting results could be attributed to the inherent limitations of the observational studies, which were prone to biases such as reverse causality and unmeasured confounding. For instance, proton pump inhibitors (PPI), a common GERD medication, could inhibit salivary secretion (Koeda et al., 2021), which consequently interferes with the assessment of oral conditions. In addition, the comorbidity of GERD and oral symptoms could also be caused by shared risk factors like smoking and drinking (Eusebi et al., 2018; Hamdi et al., 2021), rather than the existence of causality between them. To date, the association between GERD and oral symptoms has not been systematically examined, and whether GERD plays a causal role in the development of oral symptoms remains undiscerned owing to the potential biases existing in previous observational research.

Perceived as a natural analog of randomized controlled trials (RCT), Mendelian randomization (MR) leverages genetic variants as instrument variables (IVs) for exposure to explore the causal relationship between the exposure and the outcome phenotypes (Davies et al., 2018). Given that genetic variants are randomly allocated during gamete formation and conception (Thanassoulis and O'Donnell, 2009), MR is less likely to have the confounding biases and reverse causation that are common in observational studies (Davey Smith and Hemani, 2014; Skrivankova et al., 2021).

Using the MR design, several risk factors of GERD and the causal relationship between GERD and other diseases have been reported (Freuer et al., 2022; Yuan and Larsson, 2022). However, the causal association between GERD and oral symptoms has not been demonstrated yet. Mouth ulcers, toothache, loose teeth, bleeding gums, and periodontitis are the common oral symptoms related to GERD (Song et al., 2014; Deppe et al., 2015; Watanabe et al., 2017). It is not clear whether the observed association between GERD and oral symptoms in previous studies is due to causality or merely biases. To this end, we conducted a two-sample MR study, aiming to better explore the causal relationship between GERD and the five oral symptoms mentioned above.

## 2 Methods and materials

### 2.1 Data source

In this study, we obtained data on exposure (GERD) and outcome (oral symptoms) from two independent genome-wide association studies (GWAS). Data on GERD were obtained from the largest and latest GWAS conducted by Ong et al. (2022) comprising 602,604 individuals (129,080 cases and 473,524 controls). GERD cases were defined as a mixture of self-reported GERD symptoms, International Classification of Diseases diagnosis and GERD-related medication (Ong et al., 2022). The GERD data can be downloaded at IEU open GWAS project (https://gwas.mrcieu.ac.uk/datasets/).

Summary statistics for mouth ulcers, toothache, loose teeth, and bleeding gums were obtained from the UK Biobank (http://www.nealelab.is/uk-biobank/). Specifically, including mouth ulcers (47,102 cases and 414,011 controls), toothache (18,964 cases and 442,149 controls), loose teeth (18,981 cases and 442,132 controls) and bleeding gums (60,218 cases and 400,895 controls). Summary statistics for periodontitis were obtained from the Gene-Lifestyle Interactions in Dental Endpoints (GLIDE) consortium conducted by Shungin et al. (2019). The data include 45,563 European ancestry (17,353 cases and 28,210 controls).

Ethical approval was not required for this study since our analysis used publicly available GWAS summary data, and these original GWAS previously received appropriate ethics and institutional review board approval. Details of all GWASs included in our study are represented in Table 1.

**TABLE 1:** Details of the GWAS included in the Mendelian randomization.

Trait	No. of cases	No. of controls	Population	Sex	Attribute	GWAS ID/PMID
GERD	129,080	473,524	European	mix	exposure	ebi-a-GCST90000514
mouth ulcers	47,102	414,011	European	mix	outcome	ukb-b-6458
toothache	18,964	442,149	European	mix	outcome	ukb-b-19191
loose teeth	18,981	442,132	European	mix	outcome	ukb-b-12849
bleeding gums	60,218	400,895	European	mix	outcome	ukb-b-7872
periodontitis	17,353	28,210	European	mix	outcome	31235808

### 2.2 Genetic instrumental variables

MR analyses use IVs to evaluate the causal relationship between exposure (GERD) and outcome (oral symptoms). To obtain unbiased estimates, the single nucleotide polymorphisms (SNPs) selected as IVs of exposure should satisfy three key assumptions: 1) IVs used in the analysis should be significantly associated with the exposure; 2) IVs should be independent of confounders that are potentially associated with the selected exposure and outcome; 3) IVs affects the outcome only through the exposure and not *via* other biological pathways (no horizontal pleiotropic effect) (Davies et al., 2018). If all these three assumptions are met, the causal relationship between the exposure and outcome could be calculated, and unmeasured confounding and reverse causality are less likely.

**FIGURE 1:**
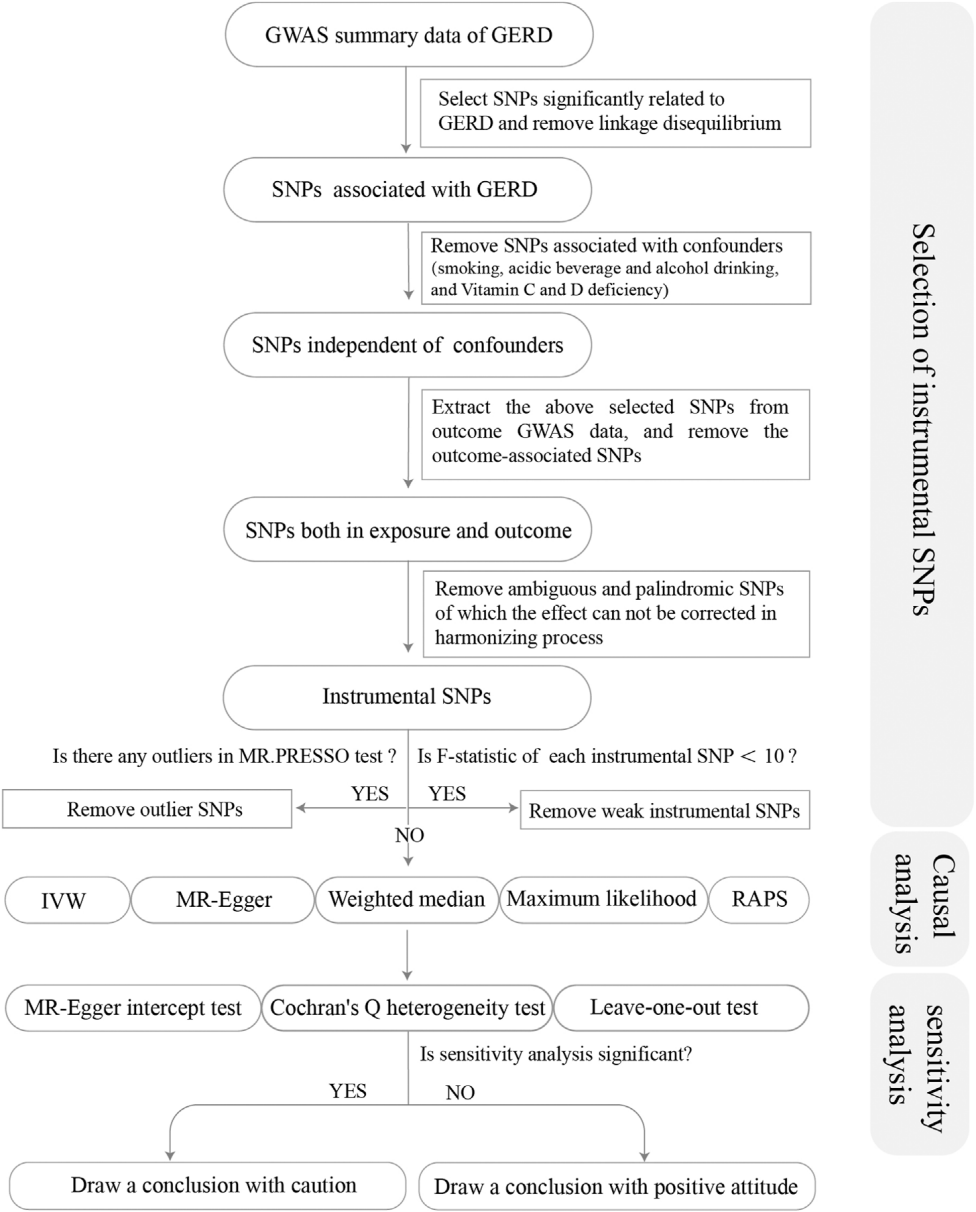
Flow chart about the analytical methods and how the MR analysis was performed step-by-step.

Note: *β* represents the genetic estimation of each SNP on the exposure; N represents sample size (number of cases plus number of controls); k represents the number of SNPs. EAF, effect allele frequency.

From the GWAS summary data of exposure, we conducted a series of quality control steps to select eligible SNPs (Figure 1). Firstly, we extracted SNPs significantly associated with the exposure (*p* < 5 × 10^–8^), and ensured that all the instrumental SNPs for the exposure were not in linkage disequilibrium (LD) (*R* ^2^ < 0.001 within 10,000 kb), since SNPs in strong LD may elicit biased results. Secondly, we removed SNPs associated with potential confounders [smoking, acidic beverage and alcohol drinking, and the deficiency of Vitamin C and D (Seong et al., 2015; Kaur et al., 2016; Hamdi et al., 2021)]. Traits related to each SNP can be found on the following website (http://www.phenoscanner.medschl.cam.ac.uk/). Thirdly, we extracted the exposure SNPs from the outcome GWAS summary data and discarded SNPs significantly associated with the outcomes (*p* < 5 × 10^–8^). Fourthly, we harmonized the exposure and outcome SNPs to keep the concordance of the effect alleles and removed the palindromic and incompatible SNPs. Fifthly, we conducted the MR-PRESSO (Pleiotropy RESidual Sum and Outlier) global test to identify inconsistencies between genetic associations of different genetic variants and remove outlying genetic variants. Finally, we calculated the *R* ^2^ and F-statistics of each SNP and the total SNPs. *R* ^2^ was calculated to represent the proportion of variance in an exposure factor explained by the IVs. F-statistic was calculated to represent the strength of the association between the instruments and exposure of interest (Burgess and Thompson, 2011). An F-statistic ≥10 for the SNPs indicated the selected SNPs were valid with sufficient strength (Burgess et al., 2011). Thereby, SNPs with F < 10 were removed from MR analysis. *R* ^2^ and F-statistics were calculated by the following equation:

$$R^{2}=2\times \left(1-EAF\right)\times EAF\times \beta^{2}$$

$$F=\left(\frac{R^{2}}{1-R^{2}}\right)\left(\frac{N-k-1}{k}\right)$$

### 2.3 Mendelian randomization analysis

We used multiple complementary MR approaches to calculate the causal relationship between GERD and oral symptoms. These approaches are random-effect inverse-variance weighted (IVW), MR-Egger regression, weighted median, maximum likelihood, and robust adjusted profile score (RAPS). We used the result of IVW as the main outcome (Burgess et al., 2013). IVW calculated a weighted average of Wald ratio for individual SNPs, which assumed that all the instruments were valid, thereby with the largest power but susceptible to biases. In this study, the random-effect model was applied for IVW as it remains conservative estimates even when heterogeneity was detected. MR-Egger regression model provided a relatively robust estimate independent of IVs validity. However, the MR-Egger method was prone to be affected by outliers, resulting in a relatively imprecise and low power (Bowden et al., 2015). The weighted median method examined the median effects of all instrumental SNPs when at least half the IVs were valid, resulting in unbiased estimates of effects (Bowden et al., 2016). Maximum likelihood was a traditional means with low standard error. It estimated the probability distribution parameters by maximizing the likelihood function. Although it may be biased with limited sample sizes, the bias was so small that it can be ignored biologically (Milligan, 2003). RAPS was a newly developed analysis, which considering the measurement error in SNP-exposure effects was conducted to reduce bias from weak IVs (Zhao et al., 2019). A Bonferroni-corrected *p*-value was set as 0.01 (0.05/five outcomes), and meanwhile *p* < 0.05 was regarded as nominally significant.

### 2.4 Sensitivity analysis

Sensitivity analysis was pivotal in MR studies to detect underlying pleiotropy and heterogeneity for MR estimates. We conducted the MR-Egger regression to assess the potential pleiotropic effects of the instrumental SNPs. The intercept term in MR-Egger regression can be a useful indication of whether directional horizontal pleiotropy was driving the results of an MR analysis. The MR-Egger intercept test with *p* < 0.05 indicated the existence of horizontal pleiotropy. As for heterogeneity, the Cochran Q test was calculated to examine the heterogeneity among different genetic variations, and Cochran Q-derived *p* < 0.05 would be regarded as considerable heterogeneity. Additionally, to test the conformity of each SNP, we performed a leave-one-out test, leaving each genetic variant one by one. If the causal relationship was still significant statistically after excluding the non-specific SNP, it provided more credible evidence for the association. The forest plot of the leave-one-out test could be found in Supplementary Figures S1–S5 for visual examination.

If all pleiotropy tests were with *p >* 0.05, and the leave-one-out test showed the MR estimates were stable, we could draw a conclusion with a positive attitude for the causal relationship between GERD and oral diseases, otherwise, we should draw a conclusion with caution.

### 2.5 Statistical analysis

All statistical tests were performed by the “TwoSampleMR” package (version 0.4.25) for the R program (version 4.1.2). The “TwoSampleMR” codes in our study were available here: https://mrcieu.github.io/TwoSampleMR.

## 3 Results

### 3.1 Genetic instruments for GERD

Based on the previously developed screening protocol, we first screened 80 SNPs from the exposure (GERD) and examined whether they were associated with potential confounders including smoking, acidic beverage and alcohol drinking, and the deficiency of Vitamin C and D (Seong et al., 2015; Kaur et al., 2016; Hamdi et al., 2021). When extracting the exposure SNPs from the outcome phenotype mouth ulcers, one SNP (rs3828917) was removed owing to a significant association with mouth ulcers (*p* = 6.50 × 10^−13^). During the process of harmonization, three SNPs (rs2145318, rs2358016, and rs957345) were removed for being palindromic with an intermediate effect allele frequency. MR-PRESSO global test found four outliers (rs1596747, rs1716171, rs7206608, and rs9940128) for mouth ulcers, 0 outliers for toothache, three outliers (rs1479405, rs1937450, and rs324769) for loose teeth, three outliers (rs10010963, rs2043539, and rs3828917) for bleeding gums and no outliers for periodontitis. Finally, 72, 77, 74, 74, and 65 SNPs remained as IVs for the above oral symptoms respectively (Table 2). F-statistics for each of the SNPs were larger than 10, which were presented in Supplementary Tables S1–S5 in detail.

**TABLE 2:** Sensitivity analysis of the causal association between GERD and oral symptoms.

Outcome	Cochran’s Q test *P*)	Intercept test *P*)	Leave-one-out test
mouth ulcers	0.201	0.921	stable
toothache	0.068	0.358	stable
loose teeth	0.134	0.449	stable
bleeding gums	0.008^*^	0.160	stable
periodontitis	0.642	0.701	stable

*Indicates that the results are statistically significant.

### 3.2 Causal estimation of GERD on oral symptoms

**FIGURE 2:**
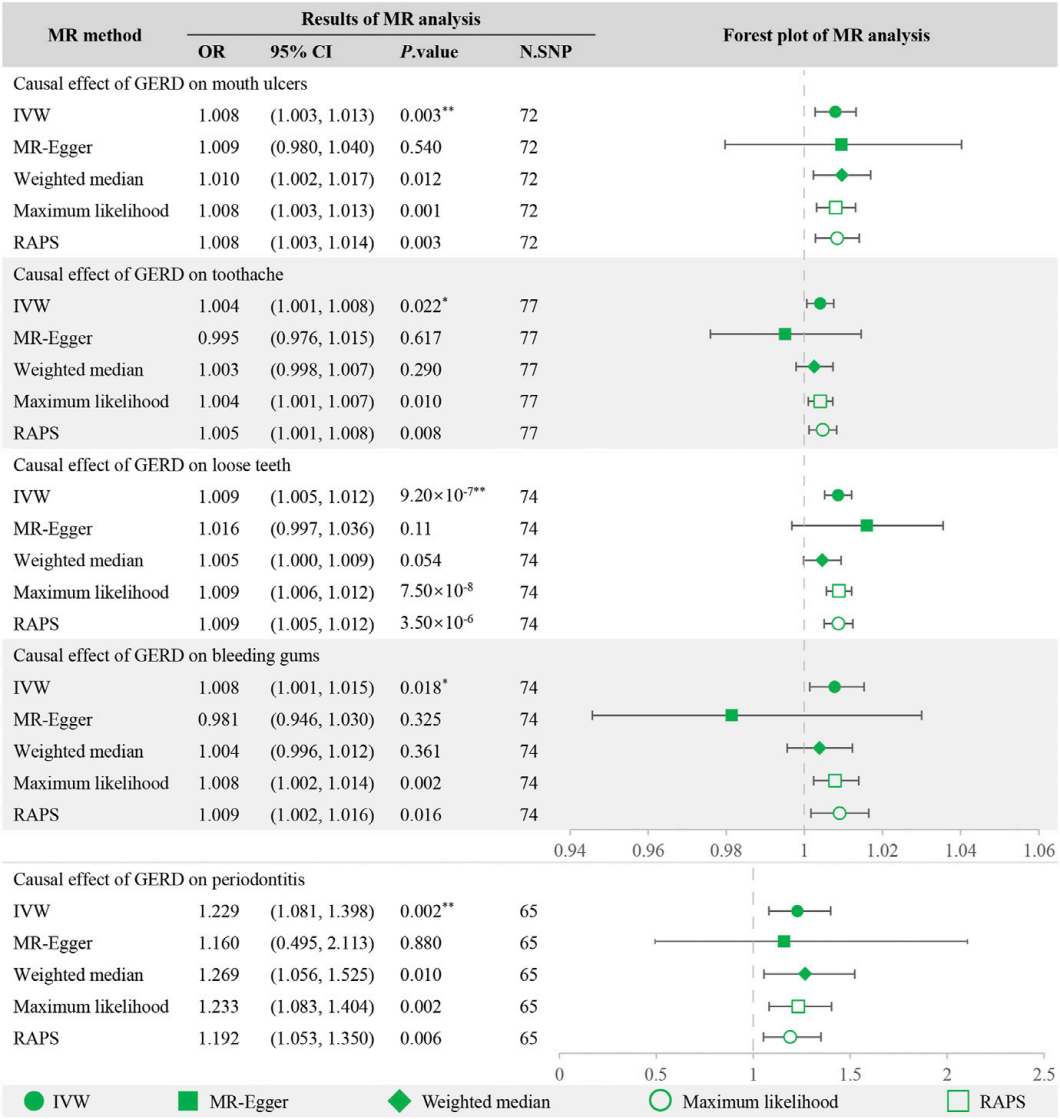
MR analysis of GERD with oral symptoms and the forest plot. **Significant estimate is defined as IVW-derived *p*. value<0.01. *Nominally significant is defined as IVW-derived *p*. value<0.05. OR, odds ratio. 95% CI, 95% confidence interval. IVW, inverse variance weighted. MR-Egger, Mendelian randomization Egger regression method. RAPS, robust adjusted profile score.

After Bonferroni correction, the IVW method detected strong evidence of the causal associations between genetic liability to GERD and an increased risk of mouth ulcers (OR = 1.008, 95% CI = 1.003–1.013, *p* = 0.003), loose teeth (OR = 1.009, 95% CI = 1.005–1.012, *p* = 9.20 × 10^−7^) and periodontitis (OR = 1.229, 95% CI = 1.081–1.398, *p* = 0.002). And for toothache (OR = 1.004, 95% CI = 1.001–1.008, *p* = 0.022) and bleeding gums (OR = 1.008, 95% CI = 1.001–1.015, *p* = 0.018), the causality seems to be nominally significant. For mouth ulcers, loose teeth, and periodontitis, MR estimations across various models, including maximum likelihood, MR RAPS, weighted median, and MR-Egger, were consistent (Figure 2), greatly enhancing the credibility of the causal inference. For toothache and bleeding gums, MR-Egger showed a discrepant direction of estimation compared with other MR methods (Figure 2), suggesting that the determination of causality should be cautious.

### 3.3 Sensitivity analysis

Table 2 summarized the results of the sensitivity analysis. Cochran Q test detected the existence of heterogeneity in the causality for bleeding gums but not in the other four oral diseases. The intercepts derived from the MR-Egger regression showed little evidence of horizontal pleiotropy. Besides, the leave-one-out analysis was stable when discarding each SNP by turns.

## 4 Discussion

We found that genetic liability to GERD was associated with an increased risk of several oral symptoms, specifically for mouth ulcers, loose teeth, and periodontitis, which may enrich our comprehension of the potential risk factors for oral symptoms among patients with GERD. To our knowledge, this is the first study to explore the causal relationship between GERD and oral symptoms using two-sample MR analysis.

Previously, results yielded from observational studies on the association between GERD and oral symptoms were inconsistent. Song et al. found that GERD was associated with an increased incidence of periodontitis (OR = 2.883; 95% CI = 1.775–4.682) (Song et al., 2014). Di Fede et al. (2008) showed that patients with GERD have no association with dental abnormality (*p* > 0.2) but have a higher risk of mucosal lesions. For mucosal lesions, Meurman et al. (1994) found no evidence of their relationship with GERD. Therefore, based on the existing evidence, the causalities between GERD and oral symptoms remain to be elucidated. Such conflicting findings could be attributable to a variety of potential biases including methods of data collection, population-specific genetic, and environmental exposures. For example, the oral symptoms in the study conducted by Meurman et al. (1994) were obtained from GERD patients during hospitalization. However, patients during hospitalization may receive better oral care and diet control, and this may explain their negative result. The population in the study conducted by Warsi et al. (2019) was Pakistanis, and the population specificity is also one of the reasons for the inconsistent conclusions. The side effects of drugs on oral cavity are often ignored in observational studies. These side effects of drugs may interfere with oral conditions (Lynge Pedersen and Belstrom, 2019; Kouznetsova et al., 2021; Watson et al., 2022). For instance, PPI could reduce salivary secretion (Koeda et al., 2021), and increase the risk of vitamin B12, calcium and iron deficiency (Insogna, 2009; Lam et al., 2013; Lam et al., 2017). In addition, most observational studies only discussed the confounding caused by acidic beverages and did not consider alcohol consumption and smoking (Meurman et al., 1994; Di Fede et al., 2008). The intake of tobacco and alcohol can lead to oral mucosa and periodontal lesions, and even oral cancer (Penteado et al., 2020). Unlike observational studies, MR analyses are less likely to be subject to confusion bias and reverse causality. Our MR analysis found positive evidence supporting a causal role of GERD on the risk of oral symptoms, including mouth ulcers, loose teeth, and periodontitis. Notably, only nominal significant effects were observed in toothache and bleeding gums, and discrepant directions were observed across distinct MR methods, and heterogeneity was detected. Therefore, affirmative conclusion for the causal effect of GERD on these two oral symptoms could not be drawn based on the present findings.

There are several mechanisms that may explain this causal relationship. First, GERD can cause oral acid-base disturbances as the regurgitated contents mainly contain gastric acid, pepsin, and sometimes may also contain bile acids and the pancreatic enzyme trypsin coming from duodenum. It has been found that the oral pH values of the patients with GERD were significantly lower than that of healthy people (Aframian et al., 2010). The endogenous acid could change the architecture of the protective enamel and dentine layers and lead to the occurrence of toothache and loose teeth (Lussi et al., 2011). Second, GERD can also decrease the oral saliva secretion and disorder the saliva buffer system (Yoshikawa et al., 2012; Bechir et al., 2021). Under normal conditions, the mucosal surface is covered with mucin-rich secretions, which form a mechanical barrier against multiple harmful factors (Dawes and Wong, 2019). Whereas under the condition of GERD, this protective effect is decreased owing to the disruption of the barrier, consequently increasing the risk of mucosal lesions. Third, GERD can cause oral microorganisms and their metabolites dysbiosis (Kawar et al., 2021). Periodontal pathogens such as *Porphyromonas gingivalis* and *Prevotella intermedia* were found significant increased in the oral cavity of patients with GERD (Sazanskaya et al., 2020). These bacterial enzymes and cytotoxic products of bacterial metabolism such as lipopolysaccharide can cause oral infection (Yousefi et al., 2020). Fourth, GERD can cause oral immune disorders. Periodontitis is a inflammatory oral disease associated with dysregulation of the innate and adaptive immune systems. Inflammatory cell infiltration was observed in the oral mucosa in rats with GERD, and the unbalance between pro-inflammatory and anti-inflammatory cytokines might involved in the development of periodontitis (Shimazu et al., 2018). These factors above can damage the mechanical, chemical, biological and immune barrier of oral cavity independently or synergistically, and consequently trigger multiple oral symptoms (Senel, 2021). Knowledge of the underlying mechanisms might be valuable for prevention and treatment of oral symptoms in patients with GERD, therefore, more studies should be conducted futher to better elucidate the mechanisms.

There are several strengths in the current study. First, the GWAS summary statistics of GERD and oral symptoms all from the largest and latest studies, and there was no overlapping sample in our study. This would greatly increase the statistical power of the causality inference. Second, we designed a rigorous screening protocol for instrumental SNPs with a large F-statistic for genetic instruments, which means that weak instrumental bias was less likely. Third, we used five complementary MR analysis methods with multiple sensitivity analysis, which reduced the false positive rate and thus ensured the accuracy of our conclusions.

Some limitations of our MR analysis should be noted. First, part of the GERD diagnosis was based on self-report, which might potentially influence the credibility of the MR result. Future studies should be performed to validate our results when GWAS data of GERD diagnosed based only on ICD criteria was publicly available. Second, the summary GWAS data were merely derived from individuals of European descent, and our results may not be fully representative of the whole population. Therefore, GWAS research involving a wider population and a more detailed GERD subtype needs to be conducted. Third, the causal effects of GERD on oral symptoms seem fairly modest in our study, suggesting that the risk of oral symptoms secondary to GERD is relatively low. However, it is reassuring that the modest MR estimates found no evidence of pleiotropy, which indicated the robustness of the MR estimates. In addition, it should also be noted that though the risk is mild as estimated in the current study, the present findings still have great implication on clinical practice considering the high prevalence of GERD.

In conclusion, this was the first MR study to explore the causality from GERD to oral symptoms. Our MR analysis demonstrated the causal effect of GERD with oral symptoms, especially for mouth ulcers, loose teeth, and periodontitis, indicating oral care should be enhanced in the population with GERD.

## Data Availability

The original contributions presented in the study are included in the article/Supplementary Material, further inquiries can be directed to the corresponding authors.
