# Supplementary material for: Gastroesophageal reflux disease and oral symptoms: A two-sample Mendelian randomization study
Source: Front Genet. 2023 Jan 4;13:1061550. doi: 10.3389/fgene.2022.1061550 (PMC9845290; doi:10.3389/fgene.2022.1061550)

**Supplementary figures of leave-one-out test.**

Figure S1. Forest plot of the result of leave-one-out test on the causual relationship between GERD and mouth ulcers.

Figure S2. Forest plot of the result of leave-one-out test on the causual relationship between GERD and toothach.

Figure S3. Forest plot of the result of leave-one-out test on the causual relationship between GERD and loose teeth.

Figure S4. Forest plot of the result of leave-one-out test on the causual relationship between GERD and bleeding gums.

Figure S5. Forest plot of the result of leave-one-out test on the causual relationship between GERD and periodontitis.

**Figure S1.**


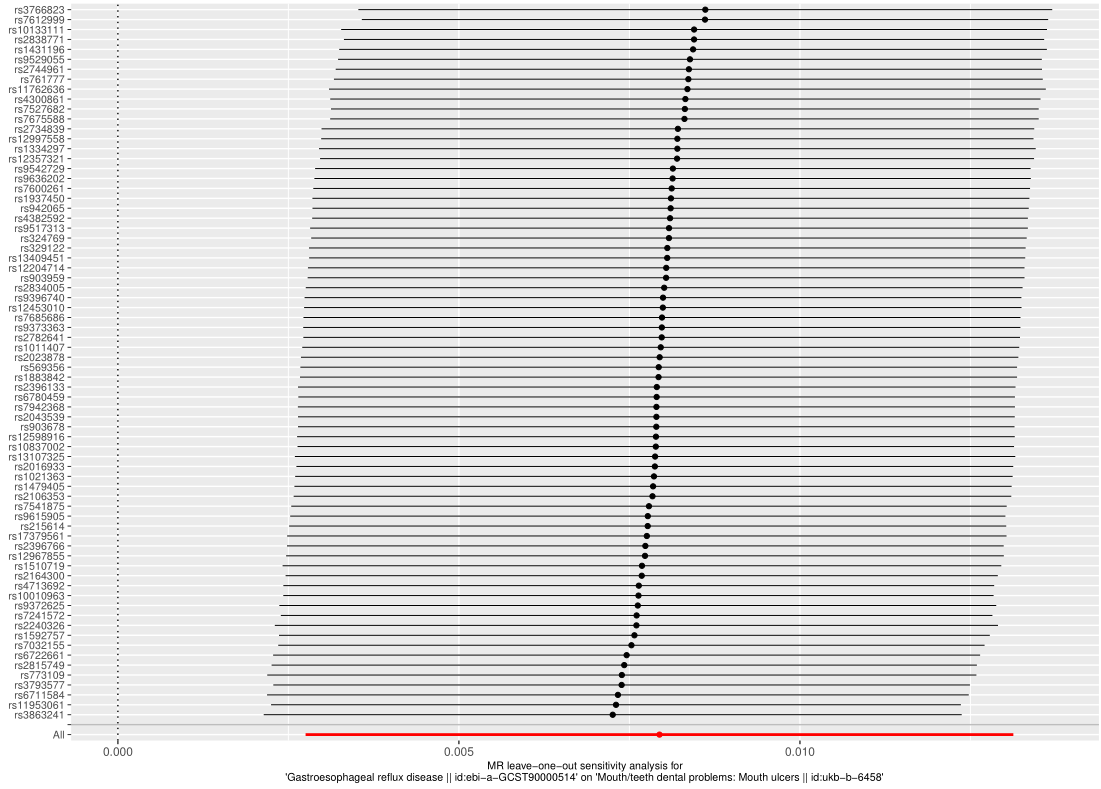


**Figure S2.**

**
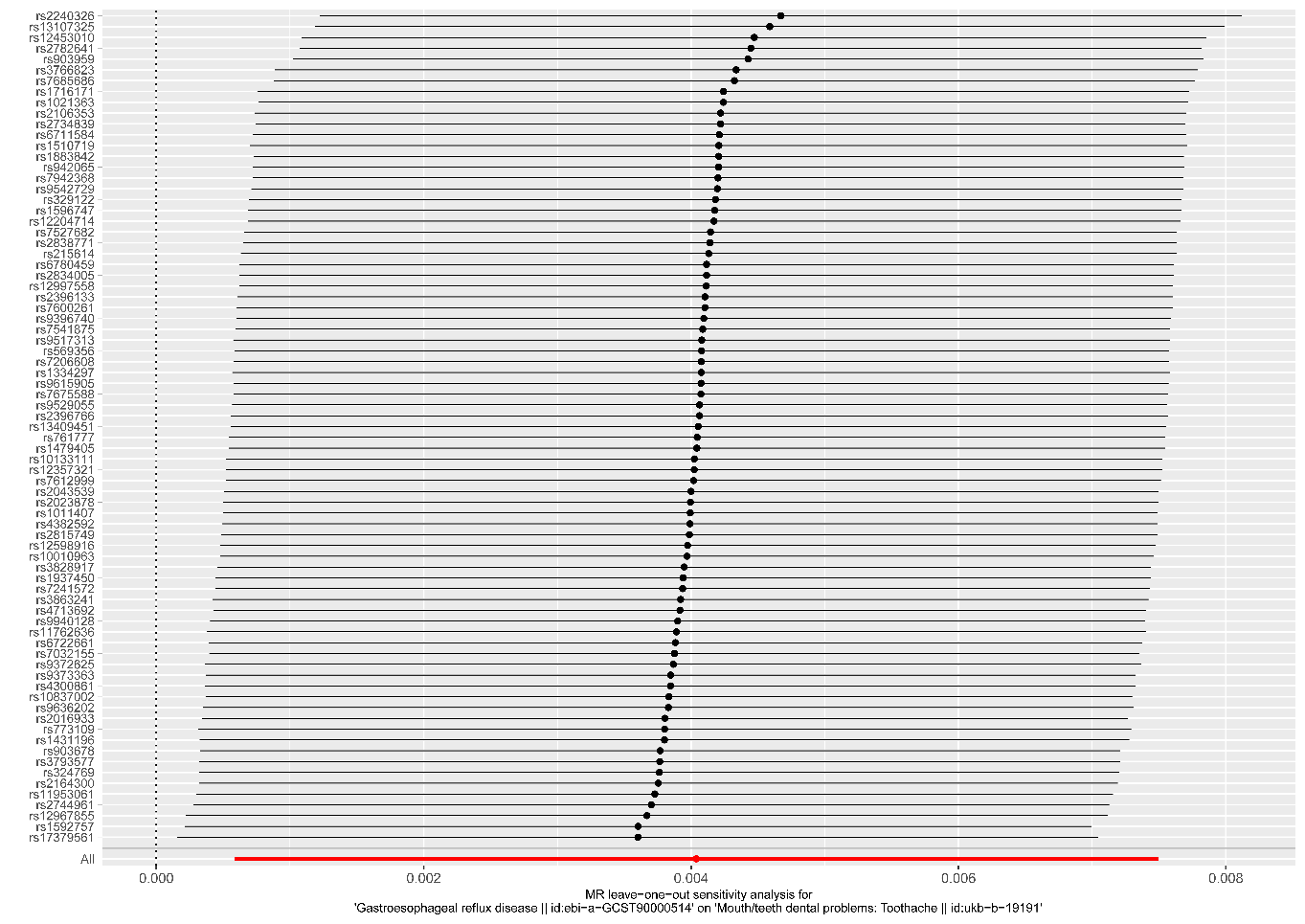
**

**Figure S3.**


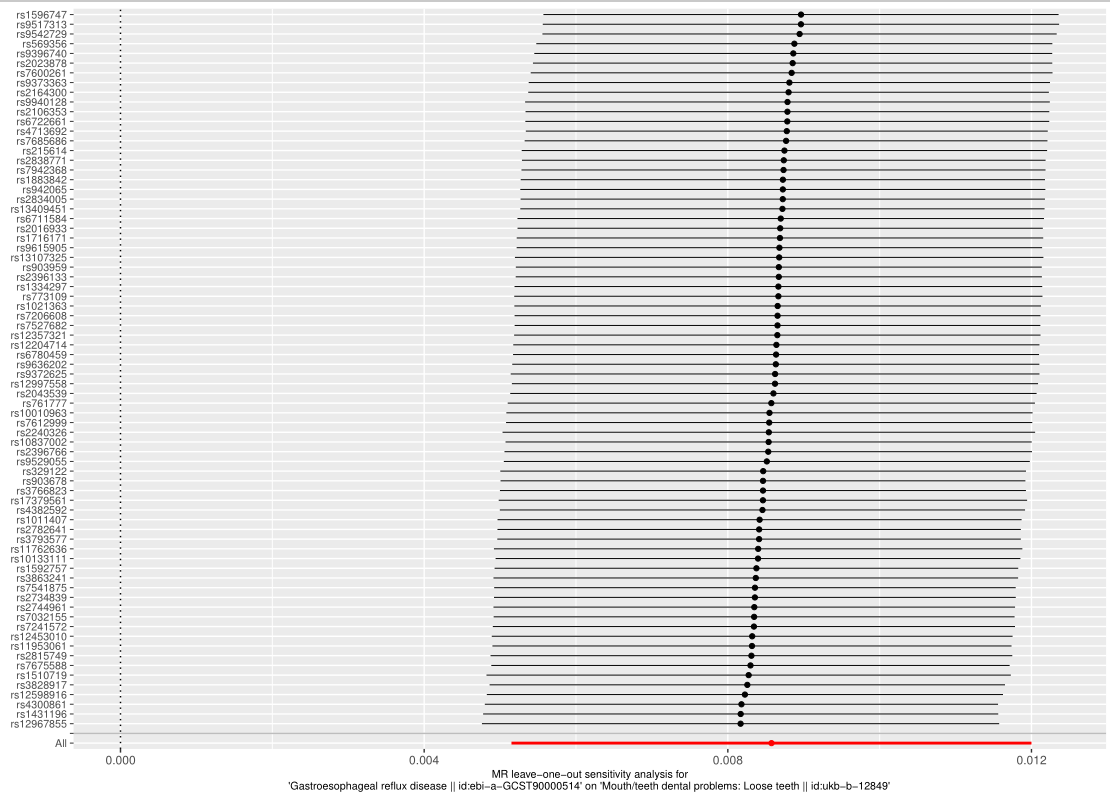


**Figure S4.**


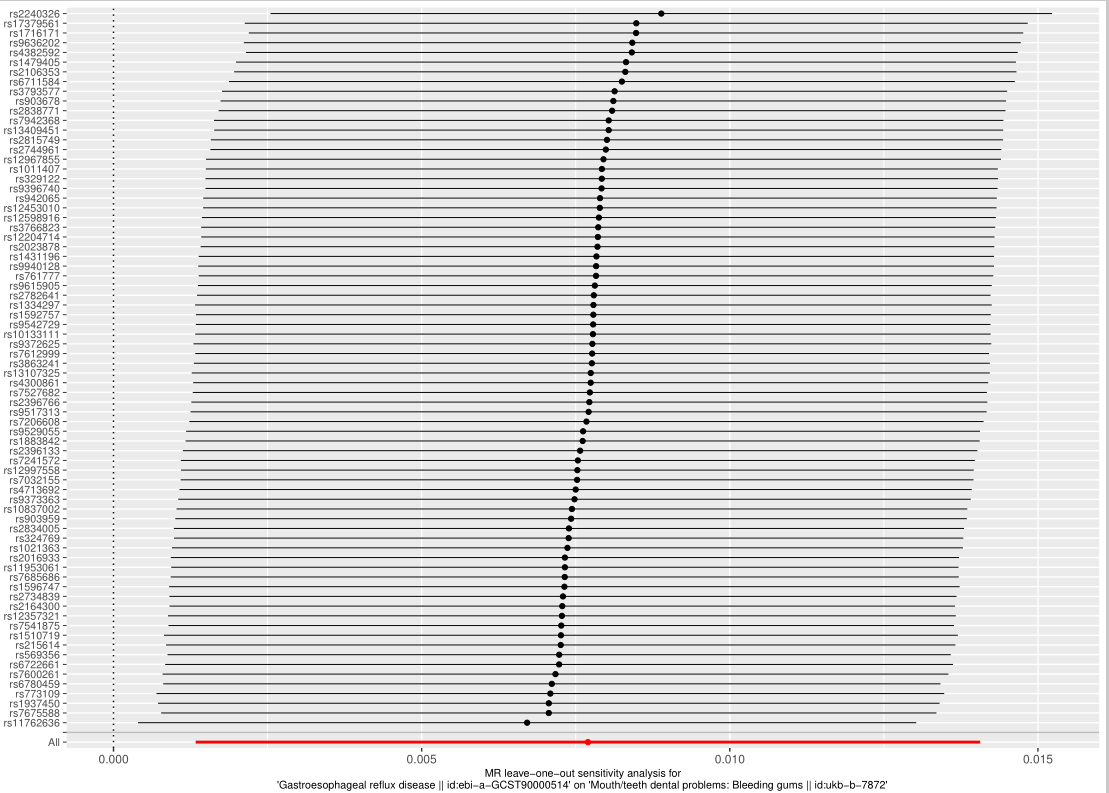


**Figure S5.**


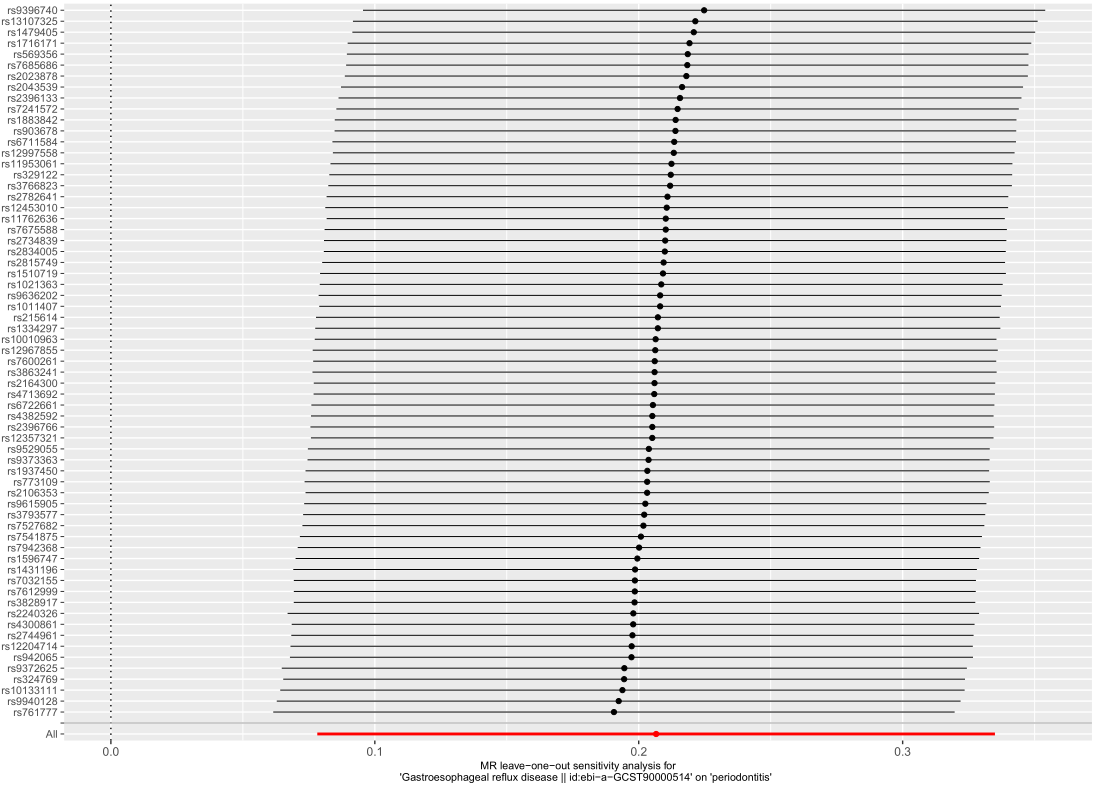

Supplement: Supplementary file 2 [file Table1.DOCX]
